# Anti TNF-Alpha Treatment Improves Microvascular Endothelial Dysfunction in Rheumatoid Arthritis Patients

**DOI:** 10.3390/ijms25189925

**Published:** 2024-09-14

**Authors:** Alexandru Caraba, Oana Stancu, Viorica Crișan, Doina Georgescu

**Affiliations:** 1 Third Internal Medicine, Diabetes and Rheumatology Department, University of Medicine and Pharmacy “Victor Babeș”, 300041 Timișoara, Romania; caraba.alexandru@umft.ro; 2 Internal Medicine Department, “Victor Popescu” Military Hospital, 300080 Timișoara, Romania; 3 Rheumatology Department, University of Medicine and Pharmacy “Victor Babeș”, 300041 Timișoara, Romania; viocrisan2002@yahoo.com; 4 Medical Semiology Department, University of Medicine and Pharmacy “Victor Babeș”, 300041 Timișoara, Romania; doina.georgescu@umft.ro

**Keywords:** anti TNF-alpha therapy, microvascular endothelial dysfunction, nailfold capillaroscopy, rheumatoid arthritis

## Abstract

Nailfold capillaroscopy is a non-invasive investigation, which allows for the study of the microvasculature (anatomical and functional). Rheumatoid arthritis (RA) is associated with a high risk of cardiovascular atherosclerotic diseases, with endothelial dysfunction (macrovascular and microvascular) representing the first step in atherosclerosis development. The aim of this study is represented by the assessment of microvascular endothelial dysfunction in RA patients by means of nailfold capillaroscopy and to assess its evolution after a period of 12 months of anti TNF-alpha treatment. The study included 70 consecutive patients with RA and 70 healthy subjects, matched for age and gender, as the control group. Rheumatoid factor, anti-cyclic citrullinated peptide antibodies, serum TNF-α, C reactive protein, and erythrocytes sedimentation rate were evaluated in all patients, but in controls, only rheumatoid factor, serum TNF-α, C reactive protein, and erythrocytes sedimentation rate were measured. The RA activity was measured by DAS28. Nailfold capillaroscopy was carried out in all patients and controls, determining the baseline nailfold capillary density (Db), nailfold capillary density during reactive hyperemia (Dh), and nailfold capillary density after venous congestion (Dc). Data were presented as mean ± standard deviation. Statistical analysis was performed using ANOVA and Pearson’s correlation, with *p* < 0.05 being statistically significant. Db, Dh, and Dc were lower in RA patients than in controls (*p* < 0.0001), correlating with RA activity and TNF-α (*p* < 0.05). After 12 months of anti TNF-α treatment, microvascular endothelial dysfunction improved (*p* < 0.0001). Microvascular endothelial dysfunction can be assessed by nailfold capillaroscopy, with anti TNF-α medication contributing to its improvement.

## 1. Introduction

Rheumatoid arthritis (RA) is an autoimmune inflammatory disorder that affects synovial joints in the form of proliferative and destructive synovitis, which impairs the quality of patients’ lives. RA affects approximately 1% of the population, inducing a significant societal and economic burden in terms of cost and disability [1,2]. Besides the articular involvement, RA has extra-musculoskeletal manifestations as well [3,4]. Extra-musculoskeletal RA manifestations are seen in about 40% of patients at some time during the disease evolution, generating a mortality risk ratio five times greater than in RA patients without these comorbidities [3,5]. Among these comorbidities, cardiovascular diseases, especially atherosclerotic ones, generate high morbidity and mortality in RA patients. It is demonstrated that the RA represents an independent risk factor for atherosclerosis [6,7]. These patients develop a premature, accelerated, and extensive atherosclerosis. The cardiovascular risk in RA patients is comparable to that reported for diabetic patients, with RA patients necessitating aggressive and targeted cardiovascular risk management [8].

Chronic and sustained inflammation represents the pathological mechanism involved in RA and atherosclerosis development [1]. Tumor necrosis factor alpha (TNF-α), interleukin-6 (IL-6), and interleukin-1 (IL-1) are some of the proinflammatory cytokines involved in RA pathogenesis. However, they have an important impact on endothelial cells as well, contributing to the initiation and perpetuation of endothelial cell activation and then dysfunction [1,9]. Endothelial dysfunction is associated with a high risk of cardiovascular events, representing the first step in atherosclerosis development [10].

Vascular endothelium represents not only a simple barrier between the bloodstream and the underlying tissues, but it is a crucial homeostatic organ, being involved in the vascular function. It acts as a vasomotor tone regulator (the balance between vasodilation and vasoconstriction), a controller of vascular permeability, hemostasis, coagulation, angiogenesis, leukocyte trafficking, and involvement in innate and adaptive immunity. In order to control the vascular tone, endothelial cells release endothelium-derived relaxing factors: nitric oxide (NO), prostacyclin, and endothelium-derived hyperpolarizing factor (EDHF) in order to counter contracting factors: vasoconstrictor prostanoids, angiotensin II, and endothelin 1 [11].

Endothelial dysfunction, found both at macrovascular and microvascular levels, is frequently identified in RA patients, even in the absence of overt atherosclerotic cardiovascular disease [12]. Macrovascular endothelial dysfunction can be assessed by means of flow-mediated dilation and serum markers (asymmetric-dimethylarginine, matrix metalloproteinase, myeloperoxidase, and toll-like receptor) [13]. Microvascular endothelial dysfunction is represented by a number of endothelium alterations: inflammatory activation, reduced capillary density, a decrease in vasodilation, and impaired angiogenesis. Reduced capillary density may be structural (a reduced number of capillaries in the tissue) and functional (decreased perfused capillaries without a reduction in their number). Nailfold capillaroscopy is a non-invasive method, which investigates microcirculation abnormalities. In rheumatology, it represents a widely used method in the diagnosis and monitoring of various rheumatic disorders [14,15,16].

In 2002, in RA patients, endothelial dysfunction was first identified by Bergholm, representing an important cardiovascular risk factor [17]. Many authors revealed the presence of endothelial dysfunction in RA patients [9,11,18,19,20]. Although endothelial dysfunction is identified at both the macrovascular and microvascular bed, there is a poor correlation between endothelial dysfunction at the microvascular and macrovascular levels, because as artery size decreases, the relative role of NO in producing endothelium-dependent vasodilatation decreases and the contribution played by EDHF increases. Microvascular endothelial dysfunction represents a good predictor for subsequent cardiovascular events in patients without pre-existing vascular involvement [21]. Microvascular dysfunction is identified from the early stage of RA, without macrovascular damage [22].

Anti TNF-α drugs, introduced in inflammatory disease therapy about two decades ago, act by counterbalancing the high TNF-α levels. TNF-α inhibitors are represented by infliximab, etanercept, adalimumab, golimumab, and certolizumab pegol. Anti TNF-α drugs reduce the inflammatory response by blocking TNF-α actions [23]. By using anti TNF-alpha medication, rheumatoid microvascular endothelial dysfunction improved, recognizing the role of proinflammatory cytokines in its production [24]. Turiel et al., using treatment with methotrexate and adalimumab, showed that the microvascular endothelial dysfunction improved, in association with a reduction in RA activity [25].

The aim of this study was represented by the assessment of microvascular endothelial dysfunction in RA patients by means of nailfold capillaroscopy and to assess its evolution during a period of 12 months of anti TNF-alpha treatment.

## 2. Results

Demographic characteristics of the RA patients and controls are presented in Table 1.

In the therapeutic regimen, all the RA patients had methotrexate (13.64 ± 3.00 mg/week), along with anti TNF-α medication.

At the time of enrollment, RA patients had an active disease; the values of ESR, C reactive protein, and TNF-α were significantly increased than in controls (Table 2).

Based on DAS28 values, the RA was classified as high disease activity (sixty-seven patients) and moderate disease activity (three patients).

At the time of enrollment in this study (T_0_), nailfold capillaroscopy revealed that the RA patients presented lower values of capillary density (Db, Dh, Dc) than the controls, being statistically significant (*p* < 0.0001). These findings are presented in Table 3.

The RA patients were treated with methotrexate and anti TNF-α drugs. After 12 months (T_12_), remission was established in all the studied patients; nailfold capillaroscopy was repeated in all of them. The obtained data are presented in Table 4.

Nailfold capillary density (Db, Dh, Dc) increased after 12 months of treatment, being statistically significant (*p* < 0.0001).

At the moment of enrollment in this study, in RA patients, capillary density was less than in controls, but after 12 months of anti TNF-α therapy, this density increased without reaching the control values (*p* < 0.0001), as seen in Table 3 and Table 4.

In RA patients, ΔDh (Dh − Db) and ΔDc (Dc − Db) were calculated at the moment T_0_. They have the values of 2.22 ± 0.95/mm^2^ and 4.44 ± 1.32/mm^2^, respectively.

The correlations between disease activity (DAS28) and capillary density (Db, Dh, Dc, ΔDh, ΔDc) are shown in Table 5, being statistically significant.

Other important correlations were identified between TNF-α values and capillary density (Db, Dh, Dc, ΔDh, ΔDc). They are presented in Table 6, being statistically significant.

It was observed that more active RA was associated with lower capillary density. Although capillary density showed statistically significant correlations with DAS28 and TNF-α, the *r* coefficient values were different. This observation is due to the fact that RA inflammation is due to both TNF-α and other mediators, and DAS28 is a composite index, which also includes a subjective component (VAS).

The RA studied patients were treated with a combination of methotrexate (13.64 ± 3.00 mg/week) and anti TNF-α drugs (infliximab: 24 patients, etanercept: 26 patients, adalimumab: 20 patients). The monitored parameters are illustrated in Table 7.

As shown in Table 7, DAS28 and TNF-α had similar values at the moment T_0_ (*p* > 0.05).

At the moment T_0_, baseline capillary density (Db) recorded a similar behavior, regardless of the medication that was used. But, after 12 months of treatment (T_12_), capillary density increased, but in a different mode, depending on the anti TNF-α drug used. The lowest increase was registered in the case of patients treated with etanercept and the highest in the case of those who received adalimumab.

## 3. Discussion

The main findings of this study were represented by demonstrating the existence of microvascular endothelial dysfunction by using nailfold capillaroscopy and its improvement after anti TNF-α treatment. Capillary densities (Db, Dh, Dc) were lower in RA patients than in controls (*p* < 0.0001), correlating with RA activity and TNF-α (*p* < 0.05). After 12 months of anti TNF-α treatment, microvascular endothelial dysfunction improved (*p* < 0.0001).

RA is a rheumatologic condition with high cardiovascular risk, due to premature, accelerated, and progressive atherosclerosis. Cardiovascular atherosclerotic diseases have been described as the main cause of premature mortality in these patients [25,26,27,28]. The cardiovascular risk associated with RA is similar to that observed in various conditions such as diabetes mellitus, psoriasis, hypertension, heart and renal failure, as well as inflammatory bowel disease, liver cirrhosis, and gut dysbiosis, where endothelial dysfunction and impaired bioactivity of nitric oxide are ubiquitous [29,30]. Recent studies emphasized that even after adjustment for other cardiovascular risk factors, RA became an important independent cardiovascular risk factor [31]. Endothelial dysfunction represents the first step in atherosclerosis development [9]. Endothelial dysfunction may affect both the macrovasculature and microvasculature. Gonzales Gay et al., Prati et al., Moroni et al., and Kotani et al. reported the role of macrovascular endothelial dysfunction in cardiovascular diseases associated with RA [32,33,34,35]. However, Gutterman et al. revealed the importance of microvascular endothelial dysfunction in the pathogenesis of cardiovascular diseases [36]. The microcirculation involvement can be found in all autoimmune rheumatic diseases, including RA [22].

Microvascular endothelial dysfunction can be evaluated by nailfold capillaroscopy, because skin microcirculation is a representative and accessible vascular bed for systemic microvascular reactivity assessment. Cutaneous microcirculation is considered a mirror of systemic microcirculatory anatomy and reactivity [37,38]. Nailfold capillaroscopy, a noninvasive method used to evaluate microcirculation, provides information for multiple microcirculatory parameters: capillary density, morphology, and flow velocity [16]. In order to investigate microvascular endothelial dysfunction by nailfold capillaroscopy, only one parameter is of interest, the capillary density/mm^2^, measured in three different postures: resting baseline (continuously perfused capillaries), post-occlusive reactive hyperemia (continuously and intermittently perfused capillaries), and post-venous occlusion (maximal capillary density, represented by all capillaries structurally present, perfused, and non-perfused). Reduced capillary density post-occlusive reactive hyperemia is an important sign of impaired functional capillary recruitment and functional rarefaction, being an index of capillary dysfunction. Capillary density post-venous occlusion is the best parameter to characterize the anatomic capillary number. Reduced capillary density post-venous occlusion represents anatomical capillary rarefaction [10,14,39,40].

Based on the DAS28 values, the RA patients from this study were classified as having high disease activity (sixty-seven patients) and moderate disease activity (three patients). These patients presented endothelial dysfunction at the microvascular bed. At the time of enrollment in this study (T_0_), nailfold capillaroscopy revealed that the RA patients presented low values of capillary density—baseline nailfold capillary density (Db), nailfold capillary density during reactive hyperemia (Dh), and nailfold capillary density after venous congestion (Dc)—than the controls, being statistically significant (*p* < 0.0001), suggesting that RA is a contributing factor to the microvascular alterations. The impact of inflammation in inducing microvascular endothelial dysfunction is supported by the significant correlation between capillary density (Db, Dh, Dc) and DAS28, with respective TNF-α values.

TNF-α, a pro-inflammatory cytokine, contributes to endothelial cell activation, the expression of adhesion molecules and the receptors of pro-inflammatory cytokine induction, the synthesis of inflammatory cytokines, the generation of oxidative stress, and the promotion of injury in endothelial cells. TNF-α is involved in endothelial cell apoptosis and inhibits endothelial progenitor cell activity, generating a deficit in endothelial repair [41,42].

Microvascular endothelial dysfunction with subsequent vascular damage is generated by the interactions between chronic inflammation, autoimmune dysregulation, and traditional cardiovascular disease risk factors, which have a high prevalence. The RA patients showed both reduced capillary blood flow and reactive hyperemia [43]. Datta et al., in a pilot study, showed that in patients with active RA, skin microvascular function was more impaired than in controls (*p* < 0.00001) and the anti-inflammatory treatment improved this dysfunction (*p* < 0.00001) [44]. Galarraga et al. evaluated skin microvascular function on a group of 128 RA patients without any history of cardiovascular disease. They identified a significant negative correlation between the level of inflammation (log_10_CRP) and maximum vasodilatation (*r* = −0.209, *p* = 0.018) [45]. Using laser Doppler perfusion, Foster et al. found microvascular abnormalities in the RA patients related to the inflammatory response [46]. Sag et al. showed the reduced density of nailfold capillaries in RA patients, correlating with inflammation [47]. Studying 99 RA patients and 35 controls, Anyfanti et al. identified a reduction in nailfold capillary density (*p* < 0.001) in the first group; this reduced density was correlated with the value of the C reactive protein. Multivariate analysis showed that C reactive proteins predicted capillary rarefaction (*p* = 0.044). On the other hand, these authors showed that the reduced capillary density correlated with high cardiovascular risk (*p* = 0.03). It is important to mention that the nailfold capillary density was associated with cardiovascular risk, calculated by using the Framingham Risk Score. Authors concluded that nailfold capillaroscopy could predict cardiovascular risk in RA patients [48].

After 12 months of treatment with anti TNF-α medication, all the studied patients achieved remission (DAS28: 2.28 ± 0.72). DAS28 values were different, depending on the type of anti TNF α treatment used, however, without statistically significant differences (*p* > 0.05). Capillary density (baseline nailfold capillary density, nailfold capillary density during reactive hyperemia, and nailfold capillary density after venous congestion) increased, being statistically significant, without reaching the control values (*p* < 0.0001). The increase in capillary density was different depending on the medication that was used; the lowest increase was registered in cases of patients treated with etanercept, and the highest was registered in cases of those who received adalimumab (*p* < 0.0001). The similar results were reported by Anghel et al. in their study. The authors showed that anti TNF-alpha therapy in RA patients can reduce the incidence of structural and functional nailfold capillaroscopic abnormalities. They identified increases in capillary densities in the order of etanercept, infliximab, and adalimumab [22]. Galarraga et al. showed that in RA patients treated with anti TNF-α agents (etanercept or adalimumab), both RA activity and microvascular endothelial dysfunction improved, being statistically significant. However, the authors did not study the impact of each drug (etanercept or adalimumab) on microvascular endothelial dysfunction [49]. Mangoni et al. studied microvascular endothelial dysfunction on 868 RA patients, using the reactive hyperemia index (RHI), another marker of microvascular endothelial function. The authors reported that anti TNF-α therapy may counteract the negative effects of RA on microvascular endothelial function more than other drugs (*p* = 0.003) [12].

Microvascular endothelial dysfunction is important in predicting further cardiovascular events in RA patients. The reduced density of nailfold capillaries has been correlated with the risk of cardiovascular diseases. On the other hand, microvascular endothelial dysfunction suggests a high risk of coronary artery disease, because this dysfunction might adversely affect coronary circulation, with an increased risk of ischemic coronary events. It has been demonstrated that microvascular endothelial dysfunction can provide additional predictive risk towards cardiovascular events to that of established risk scoring systems [12,50]. Souza et al. demonstrated that microvascular dysfunction was associated with an increase in carotid intima-media thickness [51]. Tibirica et al. showed that the mean capillary density was significantly reduced in patients with cardiovascular diseases than in controls (*p* = 0.004). During post-occlusive reactive hyperemia, capillary density was also markedly reduced in patients with cardiovascular diseases than in controls (*p* = 0.0001). On the other hand, in cardiovascular patients, the nailfold capillary density was significantly reduced during post-occlusive reactive hyperemia (*p* = 0.0073) [50]. Turiel et al. showed in 2009 that the coronary flow reserve, a marker of microvascular dysfunction, was significantly reduced in the RA patients compared with the healthy controls (*p* < 0.01). The authors identified a negative correlation between coronary flow reserve and carotid intima-media thickness (*p* < 0.05) [52]. Similar results were reported by other researchers in RA patients, suggesting the role of chronic inflammation in microvascular endothelial dysfunction appearance [53,54,55].

An important limitation of this study was the small number of patients. They were collected from a restricted area of the country (only from Timiș County). Another limitation of this study was represented by the fact that the patients with diabetes mellitus, uncontrolled hypertension, chronic kidney disease, as well as dyslipidemia were excluded, wanting to highlight only the impact of the inflammation that characterizes RA on endothelial function. On the other hand, the values of TNF-α were not determined after 12 months of anti TNF-α treatment. It is necessary to continue this study by recruiting a larger number of patients.

## 4. Material and Methods

### 4.1. Study Design

This study was an observational, retrospective one that was performed in the Department of Rheumatology, Railway Hospital, Timisoara, Romania, between August 2019 and May 2024. The study was conducted in accordance with the Declaration of Helsinki, and the study protocol was approved by the Ethical Committee of the Railway Hospital in Timisoara (no. 158/2019).

### 4.2. Patient Enrollment

The study included 70 consecutive patients with RA and 70 healthy subjects, matched for age and gender, which represented the control group. All the RA patients fulfilled the 2010 ACR/EULAR Classification Criteria for RA [26,55].

Exclusion criteria were as follows: aged under 18 years, inability to give informed consent, patients with clinical manifestations of atherosclerosis, patients with visible trauma or cosmetic procedures of the fingers, patients with other types of arthritis than RA, acute inflammatory diseases in 30 days before the investigation, pregnant or breastfeeding women, chronic kidney disease (eRFG < 60 mL/min/1.73 m^2^), dyslipidemia (total cholesterol ≥ 200 mg/dL, triglycerides ≥ 150 mg/dL), uncontrolled arterial hypertension (blood pressure ≥ 130/80 mmHg), and diabetes mellitus.

The control group consisted of healthy subjects admitted to the Internal Medicine Department, Railway Hospital, Timișoara, Romania for regular medical visits. All the RA patients and controls gave their written informed consent before enrolling in this study.

Medical history and current drug treatments (only in the case of RA patients) were documented for all the participants in this study. Complete clinical examination was performed for all the RA patients and the control group.

### 4.3. Investigated Laboratory Parameters

Rheumatoid factor (RF), anti-cyclic citrullinated peptide antibodies (anti-CCP abs), serum TNF-α, C reactive protein (CRP), and erythrocytes sedimentation rate (ESR) were evaluated in all RA patients. Serum TNF-α, CRP, ESR, and RF were evaluated in the control group as well.

The rheumatoid factor and anti-cyclic citrullinated peptide antibodies were detected by latex agglutination test, respectively, by an immunoenzymatic assay with fluorimetric detection. TNF-α levels were evaluated using the chemiluminescence method (CLIA-serum, limit of detection 2.9 pg/mL). The C reactive protein values were evaluated by the latex-immunoturbidimetry method, and ESR by the Westergren method.

### 4.4. Rheumatoid Arthritis Activity

RA activity was evaluated using Disease Activity Score 28-joint count (DAS28) (www.4s-dawn.com; accessed on 14 August 2019 and 20 May 2024), based on the number of tender and swollen joints, the values of ESR, and general health assessment (VAS). Based on the DAS28 values, the RA patients were classified as high disease activity (DAS28 ≥ 5.1), moderate disease activity (3.2 ˂ DAS28 ˂ 5.1), low disease activity (2.6 ˂ DAS28 ≤ 3.2), and remission (DAS28 ≤ 2.6) [27].

### 4.5. Nailfold Capillaroscopy

This procedure was carried out using a Dino-Lite USB digital microscope with 2.0-megapixel digital camera (Dino-Lite, ANMO Electronics Corporation, Manuscriptstraat 12-14-1321 NN, Almere, The Netherlands, magnification 200×). The procedure was performed by two independent investigators (A.C. and V.C.); the interclass correlation coefficient was 0.9526.

Nailfold capillaroscopy was performed at the time of enrollment of RA patients and controls, and then after 12 months of treatment with anti-TNF-alpha medication (only in the case of RA patients) with a cold light source. Before performing this investigation, the patients and controls took place in a room with a stable temperature of 20–22 °C for at least 15 min. They were asked to avoid smoking and drinking coffee for 4 h prior the examination. Cosmetic procedures on nails and the removal of fingernail cuticles were stopped four weeks before the procedure.

A skin area of 1 mm^2^ was selected, approximately 4.5 mm near the terminal capillaries line, in the middle of the nailfold of the third and fourth fingers of the right hand. Capillary density in the selected area was recorded for 15 s in the following conditions:

- baseline nailfold capillary density (Db);
- nailfold capillary density during reactive hyperemia (Dh);
- nailfold capillary density after venous congestion (Dc).

Reactive post-occlusive hyperemia was evaluated by placing a small cuff at the base of the studied finger, which was inflated to 260 mmHg for 4 min and then deflated, and during the 15 s following deflation, the number of capillaries (continuously and intermittently perfused) was recorded. The increase in capillary density during reactive post-occlusive hyperemia (ΔDh = Dh − Db) was calculated. In order to assess the nailfold capillary density after venous congestion, the same cuff was inflated to 60 mmHg for 2 min and then deflated, and during the 15 s following deflation, the number of capillaries (continuously and intermittently perfused) was recorded again. The capillary density increase after venous congestion was calculated using the formula ΔDc = Dc − Db) [10].

### 4.6. Statistical Analysis

A database that included anthropometric data, inflammatory markers, and disease activity scores, alongside results from nailfold capillaroscopy was created.

All the data were analyzed using the SPSS statistical software package, version 27 (IBM Corp, Armonk, NY, USA).

In order to assess the normal distribution of obtained data, the Kolmogorov–Smirnov test was used. The resulting data were presented as mean ± standard deviation. Parametric tests, such as ANOVA and Pearson’s correlation, were used for statistical analysis. All the results were presented as correlation coefficients and significance levels. The statistical significance for this study was considered to be achieved if the value of *p* was under 0.05.

## 5. Conclusions

In conclusion, microvascular endothelial dysfunction is present in RA patients, correlating with the RA activity. Nailfold capillaroscopy represents a non-invasive, repeatable method for microvascular endothelial dysfunction assessment. Treatment with anti TNF-α medication improves the microvascular endothelial dysfunction, reducing the cardiovascular risk in these patients.

## Figures and Tables

**Table 1:** Demographic characteristics of RA patients and controls.

Parameter	Value (Mean ± Standard Deviation)	
	RA Patients	Controls
Gender [n (%)] Males Females	70 32 (45.71%) 38 (54.28%)	70 32 (45.71%) 38 (54.28%)
Mean age (years)	57.58 ± 8.57	56.72 ± 8.14
Mean length of RA evolution (years)	12.47 ± 9.61	-
The immunosuppressive drugs used by the RA patients	Methotrexate (70 patients) Infliximab (24 patients) Etanercept (26 patients) Adalimumab (20 patients)	-

**Table 2:** The values of inflammatory parameters in RA patients and controls.

Parameter	Value (Mean ± Standard Deviation)		*p*
	RA Patients	Controls	
ESR (mm/h)	73.85 ± 18.30	8.45 ± 2.97	<0.0001
C reactive protein (mg/L)	60.22 ± 27.64	2.88 ± 0.98	<0.0001
TNF-alpha (pg/mL)	89.65 ± 21.25	3.76 ± 1.63	<0.0001
Rheumatoid factor (u/mL)	391.32 ± 382.31	6.65 ± 3.29	<0.0001
Anti-CCP abs (u/mL)	838.37 ±102.48	-	-
DAS28	6.51 ± 0.81	-	-

**Table 3:** Capillary density in RA patients and controls.

Parameter	RA (T_0_)	Controls	*p*
Db (number/mm^2^)	30.08 ± 1.70	35.11 ± 1.71	<0.0001
Dh (number/mm^2^)	32.3 ± 2.27	40.81 ± 1.98	<0.0001
Dc (number/mm^2^)	34.57 ± 2.41	42.78 ± 2.22	<0.0001

**Table 4:** Capillary density in RA patients.

Parameter	RA (T_0_)	RA (T_12_)	*p*
DAS28	6.51 ± 0.81	2.28 ± 0.72	<0.0001
Db (number/mm^2^)	30.08 ± 1.70	32.84 ± 1.91	<0.0001
Dh (number/mm^2^)	32.3 ± 2.27	35.41 ± 2.19	<0.0001
Dc (number/mm^2^)	34.57 ± 2.41	39.2 ± 2.80	<0.0001

**Table 5:** Correlations between DAS28 and capillary density.

Correlation between DAS28 and:	*r*	*p*
Db	−0.5602	<0.0001
Dh	−0.5155	<0.0001
Dc	−0.4917	<0.0001
ΔDh (Dh − Dc)	−0.2884	<0.05
ΔDc (Dc − Db)	−0.2734	<0.05

**Table 6:** Correlations between TNF-α and capillary density.

Correlation between TNF-α and:	*r*	*p*
Db	−0.3593	<0.01
Dh	−0.3850	<0.01
Dc	−0.3536	<0.01
ΔDh (Dh − Db)	−0.2883	<0.05
ΔDc (Dc − Db)	−0.2990	<0.05

**Table 7:** Monitored parameters in RA patients at T_0_ and T_12_ moments.

Parameter	Infliximab (24 Patients)	Etanercept (26 Patients)	Adalimumab (20 Patients)	*p*
DAS28(T_0_)	6.51 ± 0.64	6.51 ± 1	6.51 ± 0.74	>0.05
DAS28 (T_12_)	2.32 ± 0.48	2.39 ± 0.34	2.21 ± 0.51	>0.05
TNF-α	89.37 ± 22.78	89.35 ± 17.15	90.39 ± 24.99	>0.05
Db (T_0_)	30.66 ± 1.83	29.69 ± 1.84	29.9 ± 1.11	>0.05
Db (T_12_)	33.41 ± 1.31	31.46 ± 1.96	33.95 ± 1.35	<0.0001
ΔDb (T_12_ − T_0_)	2.75 ± 1.22	1.76 ± 0.58	4.05 ± 0.75	<0.0001
Dh (T_0_)	31.55 ± 1.39	31.88 ± 2.16	33.37 ± 2.63	<0.05
Dh (T_12_)	36.4 ± 1.81	33.88 ± 2.03	36.33 ± 1.57	<0.0001
ΔDh (T_12_ − T_0_)	2.4 ± 0.68	2.38 ± 0.63	2.91 ± 0.82	<0.0001
Dc (T_0_)	35.91 ± 2.73	34.19 ± 2.31	35.95 ± 1.14	<0.001
Dc (T_12_)	40.58 ± 1.63	36.34 ± 1.91	41.25 ± 1.55	<0.0001
ΔDc (T_12_ − T_0_)	4.91 ± 1.86	2.19 ± 0.69	7.8 ± 1.67	<0.0001

## Data Availability

Data is contained within the article.
